# Imaging Biomarkers for Neurodegeneration in Presymptomatic Familial Frontotemporal Lobar Degeneration

**DOI:** 10.3389/fneur.2020.00080

**Published:** 2020-02-28

**Authors:** Qin Chen, Kejal Kantarci

**Affiliations:** ^1^Department of Neurology, West China Hospital of Sichuan University, Chengdu, China; ^2^Department of Radiology, Mayo Clinic, Rochester, MN, United States

**Keywords:** imaging biomarker, presymptomatic, familial frontotemporal lobar degeneration, *MAPT*, *GRN*, *C9orf72*

## Abstract

Frontotemporal lobar degeneration (FTLD) is a neurodegenerative disorder characterized by behavioral changes, language abnormality, as well as executive function deficits and motor impairment. In about 30–50% of FTLD patients, an autosomal dominant pattern of inheritance was found with major mutations in the *MAPT, GRN*, and the *C9orf72* repeat expansion. These mutations could lead to neurodegenerative pathology years before clinical symptoms onset. With potential disease-modifying treatments that are under development, non-invasive biomarkers that help determine the early brain changes in presymptomatic FTLD patients will be critical for tracking disease progression and enrolling the right participants into the clinical trials at the right time during the disease course. In recent years, there is increasing evidence that a number of imaging biomarkers show the abnormalities during the presymptomatic stage. Imaging biomarkers of presymptomatic familial FTLD may provide insight into the underlying neurodegenerative process years before symptom onset. Structural magnetic resonance imaging (MRI) has demonstrated cortical degeneration with a mutation-specific neurodegeneration pattern years before onset of clinical symptoms in presymptomatic familial FTLD mutation carriers. In addition, diffusion tensor imaging (DTI) has shown the loss of white matter microstructural integrity in the presymptomatic stage of familial FTLD. Furthermore, proton magnetic resonance spectroscopy (^1^H MRS), which provides a non-invasive measurement of brain biochemistry, has identified early neurochemical abnormalities in presymptomatic *MAPT* mutation carriers. Positron emission tomography (PET) imaging with [^18^F]-fluorodeoxyglucose (FDG) has demonstrated the glucose hypometabolism in the presymptomatic stage of familial FTLD. Also, a novel PET ligand, ^18^F-AV-1451, has been used in this group to evaluate tau deposition in the brain. Promising imaging biomarkers for presymptomatic familial FTLD have been identified and assessed for specificity and sensitivity for accurate prediction of symptom onset and tracking disease progression during the presymptomatic stage when clinical measures are not useful. Furthermore, identifying imaging biomarkers for the presymptomatic stage is important for the design of disease-modifying trials. We review the recent progress in imaging biomarkers of the presymptomatic phase of familial FTLD and discuss the imaging techniques and analysis methods, with a focus on the potential implication of these imaging techniques and their utility in specific mutation types.

## Introduction

Frontotemporal lobar degeneration (FTLD) is a progressive neurodegenerative disorder characterized by behavioral abnormalities, language impairment, impaired social cognition, and executive function, as well as progressive supranuclear palsy (PSP), corticobasal syndrome (CBS), or motor neuron disease (1). FTLD has a strong genetic background with an autosomal dominant pattern of inheritance in about 30–50% FTLD patients (2) with major mutations in the microtubule-associated protein tau gene (*MAPT*) (3), progranulin (*GRN*), and the repeat expansions in the chromosome 9 open reading frame 72 gene (*C9orf72*).

Mutations in the gene encoding the microtubule-associated protein tau (*MAPT*) on chromosome 17 was first reported in 1998 (4) and have been found in many kindreds with familial FTLD. The vast majority of known mutations occurring in the coding region are in the repeats, causing the reduced ability of the mutant tau proteins to interact with microtubules, leading to hyperphosphorylated tau accumulation in glia and neurons causing neurodegeneration, white-matter integrity alterations, and brain atrophy years before clinical symptom onset (3, 5–8). Subtypes of *MAPT* mutations were associated with different types of tauopathies. These are mutations inside exon 10 (i.e., N279K, S305N, and P301L) and outside exon 10 (i.e., R406W and V337M). Mutations inside exon 10 tend to form four tandem microtubule-binding domain repeat (4R-tau) pathology rather than 3 repeat (3R-tau) pathology (9), while mutations outside exon 10 tend to form mixed 3R/4R tau pathology.

In 2006, loss-of-function mutations in the progranulin gene (*GRN*) were first reported to cause familial FTLD (10, 11). There are more than 70 different pathogenetic *GRN* mutations that have been identified until this date (http://www.molgen.ua.ac.be/FTDmutations). Mutations in the *GRN* gene leads to a loss of progranulin levels through haploinsufficiency, and the intraneuronal aggregation of TAR DNA-binding protein (TDP)-43 protein (12). The most frequent clinical phenotypes of *GRN* mutation carriers were behavioral variant frontotemporal dementia (bvFTD), CBS, and primary progressive aphasia (PPA) (13, 14). *GRN* mutation carriers have FTLD with TDP-43 inclusions and present with a diverse clinical phenotype and a highly heterogeneous age of disease onset.

In 2011, the hexanucleotide GGGGCC (G4C2) repeat expansions of the chromosome 9 open reading frame 72 gene (*C9orf72*) *C9orf72* gene were found as a common cause of both FTD and amyotrophic lateral sclerosis (ALS) (15, 16). The most common clinical phenotypes associated with *C9orf72* expansions are bvFTD (17), ALS, or the combination of both in one person (18). Cases with the *C9orf72* repeat expansion with histopathological correlation had TDP-43 depositions (18).

These mutations associated with familial FTLD could lead to brain neurodegeneration years before symptom onset. Investigation of families with the presence of *MAPT, GRN*, or *C9orf72* provide a unique opportunity to shed light on early neurodegenerative changes and also identify biomarkers for tracking disease progression in future disease-modifying trials. In recent years, there is growing evidence that a number of imaging biomarkers show abnormalities during the presymptomatic stage. Imaging biomarkers of presymptomatic familial FTLD may provide insight into the underlying neurodegenerative process years before symptom onset.

## Discussion

### Structural MRI

Structural magnetic resonance imaging (sMRI) has captured cortical degeneration with a mutation-specific neurodegeneration pattern years before onset of clinical symptoms in presymptomatic familial FTLD mutation carriers (6, 19–32). Studies of sMRI using different analysis methods, such as region of interest (ROI) in specific brain regions, cortical thickness analysis and voxel-based morphometry (VBM), could capture the gray matter and white matter volumes, cortical thickness, and the subcortical gray matter volume.

#### *MAPT*_sMRI

Previous cross-sectional MRI studies demonstrated atrophy in the anteromedial temporal lobe and orbitofrontal cortex in asymptomatic *MAPT* mutation carriers (6, 19, 20), while others found no difference between asymptomatic *MAPT* mutation carriers and controls (6, 7, 33). Recently, two longitudinal studies from a cohort of asymptomatic *MAPT* mutation carriers have reported that hippocampal volumes decline during a 2-year follow-up, but no cortical atrophy was found in longitudinal analysis with 4 years of follow-up (21, 22). During a 10-year follow-up, the rates of temporal lobe atrophy were accelerated in asymptomatic *MAPT* mutation carriers who were asymptomatic during follow-up, while accelerated atrophy rates in the temporal, parietal and frontal lobes were reported in *MAPT* mutation carriers who became symptomatic compared to non-carriers (23). These data altogether suggest the consistent finding of early involvement of the anterior–medial temporal lobe in asymptomatic *MAPT* mutation carriers. Taken together, the cross-sectional and longitudinal studies of lobar cortical atrophy in *MAPT* mutation carriers suggest a sequential pattern throughout the disease course. The cortical volume appears to decline in the temporal lobe early in the asymptomatic stage, with an acceleration of atrophy rates along with development of symptoms, followed by the frontal and parietal lobe atrophy with sparing of the occipital lobe (23) (Table 1).

**Table 1:** Studies investigating asymptomatic *MAPT* mutation vs. controls.

**No**.	**Author**	**Year**	**Study design**	**No. of subjects**	**Techniques**	**Findings**
1	Miyoshi et al. (34)	2010	Cross-sectional	3 a*MAPT*+ vs. 9 HC	[^11^C] DAA1106 PET	Glial activities were increased in the frontal cortex of 1 a*MAPT+*, the occipital cortex of 2 a*MAPT*+, and the posterior cingulate cortex of 1 a*MAPT*+
					[^11^C]dopa PET	Low dopamine synthesis in putamen
					[^11^C] MP4A PET	Reduced AChE activity in the temporal, parietal cortex
2	Kantarci et al. (8)	2010	Cross-sectional	14 a*MAPT*+ vs. 24 HC	^1^H MRS	Elevated mI/Cr and decreased NAA/mI in PCC voxel
3	Whitwell et al. (33)	2011	Cross-sectional	8 a*MAPT*+ vs. 8 NC	sMRI	No difference
					rfMRI	Reduced connectivity in the DMN
4	Dopper et al. (7)	2014	Cross-sectional	11 a*MAPT*+ vs. 8 NC	sMRI	No difference
					DTI	Decreased FA and increased RD in bilateral uncinate fasciculi; and reduced FA in the forceps minor
					rfMRI	No difference
5	Rohrer et al. (19)	2015	Cross-sectional	15 a*MAPT*+ vs. 8 NC	sMRI	Atrophy in the hippocampus, amygdala, temporal lobe, and insula
6	Dopper et al. (35)	2016	Longitudinal	11 a*MAPT*+ vs. 31 NC	ASL	No difference
7	Fumagalli et al. (20)	2018	Cross-sectional	24 a*MAPT*+ vs. 148 NC	sMRI	No difference
8	Cash et al. (6)	2018	Cross-sectional	23 a*MAPT*+ vs. 144 NC	sMRI	No difference
9	Jiskoot et al. (36)	2018	Cross-sectional	17 a*MAPT*+ vs. 115 NC	DTI	Reduced FA and increased diffusivity in the uncinate fasciculus and cingulum
10	Jones et al. (37)	2018	Cross-sectional	3 a*MAPT*+ vs. 241 HC	^18^F-AV-1451 tau PET	Low level of uptake in 1 asymptomatic N279K mutation carrier; little to no signal in 1 R406W mutation carrier, high uptake in 1 R406W mutation carrier
11	Panman et al. (22)	2019	Longitudinal	14 a*MAPT*+ vs. 53 NC	sMRI	Baseline: No difference Follow-up: lower GM volume in the left temporal pole, a trend toward cortical thinning of the right inferior temporal lobe; Longitudinal: GM volume decline in the hippocampus
				14 a*MAPT*+ vs. 50 NC	DTI	No difference
12	Chen et al. (38)	2019	Longitudinal	12 a*MAPT*+ vs. 20 NC	DTI	Baseline: higher MD in entorhinal WM
				10 a*MAPT*+ vs. 10 NC		Longitudinal: accelerated annualized change of entorhinal MD
13	Chen et al. (23)	2019	Longitudinal	14 a*MAPT*+ (include 4 converters) vs. 23 NC	sMRI	Faster rates of atrophy in temporal lobe in a*MAPT*+Increased atrophy rates in the temporal, frontal and parietal lobes in *MAPT*+ converters
14	Chen et al. (39)	2019	Cross-sectional	9 a*MAPT*+ vs. 25 NC	^1^H MRS	Lower NAA/Cr and lower NAA/mI in the frontal lobe
15	Chen et al. (40)	2019	Longitudinal	8 *MAPT*+ converters	^1^H MRS	NAA/mI ratio decreasing and mI/Cr ratio increasing accelerated 2 years before symptom onset

**Figure 1:**
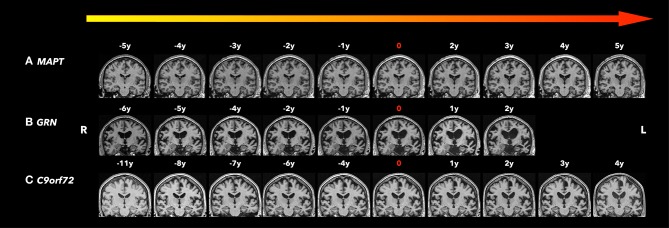
Coronal T1-weighted magnetic resonance imaging (MRI). Brain MRIs (coronal T1-weighted images) of the three familiar FTLD mutation carriers who progressed from the asymptomatic to the symptomatic stage are shown in the timeline with years before and after conversion. 0 indicates the actual symptom onset time point. **(A)** A male patient with *MAPT* N279K mutation whose first clinical manifestation was difficulties in remembering street names and people's names with Kokmen short test of mental status (36/38) at age 43. Two years after symptom onset, his forgetfulness progressed, with resting tremor in the left lower limbs, as well as some impulsivity and disinhibition, and was diagnosed with bvFTD and Parkinsonism. Five years after symptom onset, his cognitive function, parkinsonism, and behavior changes significantly worsened and mostly by motor impairment. Focal adjacent mesial temporal lobe atrophy mainly in the right side was observed 5 years before symptom onset and became bilateral hippocampal atrophy with generalized cerebral atrophy most marked in the bilateral parietotemporal regions. The structural brain change seems to be slower than his clinic symptom progression. **(B)** A male patient *GRN* mutation carrier whose first clinical manifestation was word-finding difficulties at age 68. Two years after symptom onset, his word-finding difficulties progressed, with additional executive dysfunction, forgetfulness, and tendency to repeat questions. He also had apathy, irritability, and dysphoria, and was diagnosed with mixed PPA/bvFTD syndrome. Asymmetrical (left greater than right) atrophy was observed 6 years before symptom onset, and became progressively asymmetric affecting the frontal, temporal, and parietal lobes as he aged. **(C)** A male patient *C9orf72* mutation carrier whose first clinical manifestation was a change in motivation, spontaneity, and empathy at age 73 and was diagnosed as behavior MCI. After 4 years from symptom onset, his behavior change slightly progressed with subtleties in empathy, emotional blunting, irritability, and subtle apathy, still diagnosed as behavior MCI. While his clinic symptoms seem to be stable, the moderate parenchymal volume loss was observed 8 years before symptom onset most involved in the frontal and temporal areas, and progressed affecting the whole brain with greatest atrophy in the fronto-temporal regions as he aged.

Across different subtypes of *MAPT* mutations (IVS10+16, IVS10+3, N279K, S305N, P301L, and V337M), similar patterns of atrophy were reported in the later symptomatic phase (41). However, the atrophy pattern in the early disease phase may be varied across the different subtypes of *MAPT* mutations. For example, patients with *MAPT* N279K mutations have prominent motor symptoms early in the disease process (42–44) (Figure 1A), and therefore involvement of the primary and supplementary motor cortices may be expected in this sub-mutation group (45–49). The N279K *MAPT* mutation carriers' trajectories of lobar atrophy, such as the supplemental motor cortex involvement, may be specific to the suspected 4R-tau associated neurodegeneration in N279K kindred. Further studies are needed to characterize the trajectories of rates of cortical atrophy across the different subtypes of *MAPT* mutations.

#### *GRN*_sMRI

In FTLD patients with *GRN* mutations, the pattern of brain atrophy was reported to be asymmetric and widespread, predominantly involving the frontal, inferior parietal, and posterior temporal cortices (6, 13, 50–54). In asymptomatic *GRN* mutation carriers, the structural MRI studies were inconsistent (24–27).

Although most cross-sectional (6, 7, 20, 32, 55–57) and longitudinal structural MRI studies (22) reported no gray matter volume differences between asymptomatic *GRN* mutation carriers and controls, a few recent studies found a gray matter atrophy pattern mainly involving the frontal (25), parietal (58), and temporal lobes (27). Brain atrophy was reported in the insula early, 15 years before the expected symptom onset, followed by the temporal and parietal lobes at 10 years before the expected symptom onset, then in the striatum at 5 years before the expected symptom onset (19). In a longitudinal study of asymptomatic *GRN* mutation carriers with a 20-month follow-up, no gray matter volume loss was found at baseline, but left inferior and middle temporal gyri atrophy was reported 20 months later (26). Reduced gray matter density was also reported in bilateral orbitofrontal, anterior temporal, and insular cortices at baseline with greater annualized GM density changes in right orbitofrontal and left occipital cortices during follow-up (27). A recent study reported a sequential pattern of regional cortical atrophy rates with up to 10 years of follow-up, involving the frontal and parietal lobe cortices early during the asymptomatic stage, followed by the temporal lobe cortex after symptom onset in *GRN* mutation carriers (28) (Table 2).

**Table 2:** Studies investigating asymptomatic *GRN* mutation vs. controls.

**No**.	**Author**	**Year**	**Study design**	**No. of subjects**	**Techniques**	**Findings**
1	Borroni et al. (55)	2008	Cross-sectional	7 a*GRN*+ vs. 15 HC	sMRI	No differences
					DTI	Reduced FA in left uncinate fasciculus, left inferior occipitofrontal fascisulus
2	Borroni et al. (56)	2012	Cross-sectional	9 a*GRN*+ vs. 13 NC	sMRI	No differences
					rfMRI	Increased connectivity in SN
3	Jacova et al. (59)	2013	Cross-sectional	9 a*GRN*+ vs. 11 NC	^18^FDG-PET	Lower FDG uptake in right medial, ventral frontal cortex and insula
4	Moreno et al. (57)	2013	Cross-sectional	13 a*GRN*+ vs. 13 HC	sMRI	No difference
5	Premi et al. (60)	2013	Cross-sectional	17 a*GRN*+	rfMRI	Global reserve index was inversely related to functional activation of the ventral SN in the right precentral gyrus and of the dorsal SN in the right middle temporal gyrus
6	Dopper et al. (7)	2014	Cross-sectional	28 a*GRN+* vs. 28 NC	sMRI,	No difference
					DTI	No difference
					rfMRI	Reduced functional connectivity in SN and DMN
7	Pievani et al. (25)	2014	Cross-sectional	5 a*GRN*+ vs. 5 NC	sMRI	Atrophy in the right orbitofrontal, precentral gyrus, and left rostral middle frontal gyrus
					DTI	Increased AD in the right cingulum, superior longitudinal fasciculus, and corticospinal tract.
					rfMRI	No difference
8	Premi et al. (61)	2014	Cross-sectional	17 a*GRN*+ vs. 38 HC	rfMRI	Reduced ReHo in the left parietal region Increased ReHo in the bilateral mesial frontal cortex
9	Premi et al. (62)	2014	Cross-sectional	17 a*GRN*+ vs. 14 HC	rfMRI	Decreased brain connectivity within the left frontoparietal network and increased connectivity in the executive network The TMEM106B at-risk polymorphism (T/T) was associated with decreased connectivity within the ventral SN and the left frontoparietal network
10	Caroppo et al. (26)	2015	Longitudinal	16 a*GRN*+ vs. 17 NC (baseline)	sMRI	Baseline: no difference
				14 a*GRN*+ vs. 14 NC (follow-up)		Follow-up: atrophy in the left middle and inferior temporal gyri
					^18^FDG-PET	Baseline: hypometabolism in the left middle temporal gyrus
						Follow-up: hypometabolism in the frontal, temporal lobes and thalamus
11	Rohrer et al. (19)	2015	Cross-sectional	45 a*GRN*+ vs. 93 NC	sMRI	Atrophy in the insula (15 years before EO), then in the temporal and parietal lobes (10 years before EO) and the striatum (5 years before EO)
12	Dopper et al. (35)	2016	Longitudinal	23 a*GRN*+ vs. 31 NC	ASL	Baseline: no difference
						Follow-up: fronto-parietal hypoperfusion
						Longitudinal: stronger decrease in CBF in the frontal, temporal, parietal and subcortical regions
13	Galimberti et al. (63)	2018	Cross-sectional	19 sym *GRN*+, 64 a*GRN*+ vs. 77 NC	sMRI	No correlation of GM with plasma progranulin levels in symptomatic and asymptomatic *GRN* mutation carriers as a whole group
14	Alexander et al. (64)	2018	Cross-sectional	5 a*GRN*+ vs. 11 NC	Task-based fMRI	Lower task-evoked functional activation in the anterior and posterior ventrolateral prefrontal cortex
					sMRI	No difference
15	Cash et al. (6)	2018	Cross-sectional	65 a*GRN*+ vs. 144 NC	sMRI	No difference
16	Jiskoot et al. (36)	2018	Cross-sectional	52 a*GRN*+ vs. 115 NC	DTI	Reduced FA and increased diffusivity in the internal capsule
17	Olm et al. (27)	2018	Longitudinal	11 a*GRN*+ vs. 11 NC	sMRI	Baseline: reduced GM density in bilateral orbitofrontal, insular, and anterior temporal cortices
						Longitudinal: greater annualized GM density changes in the right orbitofrontal and left occipital cortices.
					DTI	Baseline: reduced FA in bilateral superior longitudinal fasciculus, left corticospinal tract, and frontal corpus callosum
						Longitudinal: greater annualized FA change in the right superior longitudinal fasciculus and frontal corpus callosum.
18	Gazzina et al. (58)	2018	Cross-sectional	19 a*GRN*+ vs. 17 NC	sMRI	Increased cortical thickness and decreased surface area of the right parietal lobe
19	Fumagalli et al. (20)	2018	Cross-sectional	66 a*GRN*+ vs. 148 NC	sMRI	No difference
20	Popuri et al. (32)	2018	Cross-sectional	9 a*GRN*+ vs. 37 NC	sMRI	No difference
21	Chen et al. (28)	2019	Longitudinal	8 a*GRN*+ vs. 10 NC	sMRI	Increased rates of atrophy in the frontal and parietal lobe cortices
22	Panman et al. (22)	2019	Longitudinal	33 a*GRN*+ vs. 53 NC	sMRI	No difference
				28 a*GRN*+ vs. 50 NC	DTI	No difference

These inconsistencies might be due to the age at examination, the proximity to onset of clinical symptoms, associated lobar cortical atrophy changes, the sample size of *GRN* mutation carriers, and the statistical analysis methodology with different sensitivity and specificity. For example, voxel-based analysis needs larger effect sizes and corrections for multiple comparisons to detect differences in cortical volumes between groups than the targeted ROI analysis. Furthermore, many studies have demonstrated the heterogeneity of clinical presentation during the early phase and variability in age of onset in *GRN* mutations carriers (65, 66). In addition, investigations in different time windows during the disease course may capture the variable findings in the laterality of lobar cortical atrophy across individuals with *GRN* mutations. It may partly explain the inconsistent results of cortical atrophy among the prior cross-sectional studies of asymptomatic *GRN* mutation carriers.

In *GRN* mutation carriers, asymmetric atrophy is a common finding with left or right asymmetry reported even within the same family (51, 54). Asymmetric cortical atrophy was found early, 5 years before expected symptom onset in asymptomatic *GRN* mutation carriers (19), while the underlying pathological cause for the asymmetry remains unclear. In contrast, symmetric rates of brain atrophy were also found in symptomatic *GRN* mutation carriers in a longitudinal study (67). This discrepancy suggests that asymmetry in cortical atrophy may be more predominant during the early stages, becoming more symmetric during the later stages of the disease (67). Also, as noted above, the specific phenotype and associated lobar cortical atrophy changes and the differences in analysis methods may be underlying these discrepancies. It is important to note that laterality occurs at various stages of neurodegenerative disease progression, which may increase the variability in atrophy rates in individual *GRN* mutation carriers (Figure 1B).

#### *C9orf72*_sMRI

In asymptomatic *C9orf72* mutation carriers, cross-sectional structural MRI studies consistently found a diffuse pattern of atrophy including frontal, temporal, parietal, insular, and posterior cortical regions, as well as subcortical volumes of thalamus, hippocampus, and the cerebellum (6, 22, 29–32), while very few studies reported no difference between asymptomatic *C9orf72* mutation carriers and healthy controls (20, 68). The atrophy of subcortical regions is estimated to occur as early as 25 years before the expected symptom onset in *C9orf72* mutation carriers, and later involves temporal and frontal lobes at around 20 years before the expected symptom onset, and finally involving the cerebellum at around 10 years before the expected symptom onset (19). The early involvement of subcortical regions is a specific atrophy pattern in asymptomatic *C9orf72* mutation carriers that distinguishes them from other FTLD mutation carriers.

To date, two longitudinal investigations have reported that there is no difference in the trajectory of cortical atrophy rates between asymptomatic *C9orf72* mutation carriers and controls (22, 68), suggesting that longer time follow-up duration is needed to track the trajectory in asymptomatic *C9orf72* mutation carriers (Table 3). Figure 1C shows serial structural MRIs from a *C9orf72* mutation carriers who converted from asymptomatic phase to symptomatic phase during follow up.

**Table 3:** Studies investigating asymptomatic *C9orf72* mutation vs. controls.

**No**.	**Author**	**Year**	**Study design**	**No. of subjects**	**Techniques**	**Findings**
1	Rohrer et al. (19)	2015	Cross-sectional	18 a*C9*+	sMRI	Atrophy in subcortical areas (the thalamus, insula, and posterior cortical areas) at 25 years before EO, followed by the frontal and temporal lobes at 20 years before EO, and the cerebellum at 10 years before EO
2	Walhout et al. (29)	2015	Cross-sectional	16 a*C9*+ vs. 23 NC	sMRI DTI	Cortical thinning in the temporal, parietal and occipital regions, and smaller volume in the left caudate and putamen. No difference
3	Floeter et al. (68)	2016	Longitudinal	7 a*C9*+ vs. 28 HC	sMRI	Baseline: no difference
				5 a*C9*+ vs. 23 HC		Longitudinal: no difference
4	Lee et al. (30)	2017	Cross-sectional	15 a*C9*+ vs. 67 HC	sMRI	Lower GM intensity in the bilateral posterior mid-cingulate, left medial pulvinar thalamus, and small, scattered regions in the bilateral dorsolateral prefrontal cortex
				12 a*C9*+ vs. 29 HC	DTI	Reduced FA in the corpus callosum, cingulum bundles, corticospinal tracts, uncinate fasciculi and inferior longitudinal fasciculi.
				13 a*C9*+ vs. 30 HC	rfMRI	Intrinsic connectivity deficits in DMN, sensorimotor, but most prominent in SN and medial pulvinar thalamus-seeded networks
5	Papma et al. (69)	2017	Cross-sectional	18 a*C9*+ vs. 15 NC	sMRI	No difference for whole group; in a subgroup >40 years, lower gray matter volume in the right inferior temporal gyrus, right cerebellum, left postcentral and precentral gyrus, the left superior parietal lobe and the left thalamus
					DTI	Lower FA and higher RD within the right superior corona radiata, inferior longitudinal fasciculus, uncinate fasciculus, internal and external capsule, bilateral anterior thalamic radiation and corticospinal tract
6	Bertrand et al. (31)	2018	Cross-sectional	41 a*C9*+ vs. 39 NC	sMRI	Atrophy in the frontal, inferior temporal and parietal cortex and bilateral thalamus
					DTI	Lower FA, higher diffusivity in the frontal regions and bilateral corticospinal tracts
7	Cash et al. (6)	2018	Cross-sectional	40 a*C9*+ vs. 144 NC	sMRI	GM loss bilaterally in the thalamus, right superior posterior cerebellum, superior temporal and inferior frontal regions
8	Jiskoot et al. (36)	2018	Cross-sectional	35 a*C9*+ vs. 115 NC	DTI	Posteriorly located WM tracts (posterior thalamic radiation, splenium of the corpus callosum, posterior corona radiata)
9	Fumagalli et al. (20)	2018	Cross-sectional	24 a *C9*+ vs. 148 NC	sMRI	No difference
10	Popuri et al. (32)	2018	Cross-sectional	15 a*C9*+ vs. 37 NC	sMRI	Cortical thinning in the temporal, parietal and frontal regions; reduced volumes of bilateral thalamus and left caudate
11	Panman et al. (22)	2019	Longitudinal	11 a*C9*+ vs. 53 NC	sMRI	Baseline: GM volume loss in the cerebellum, insula, left fronto-temporal lobes; cortical thinning in the right postcentral gyrus
						Follow-up: GM volume loss in the thalamus, cerebellum, and several bilateral orbitofrontal and insular cortices, and the postcentral gyrus; Cortical thinning in bilateral precentral gyrus and right superior parietal lobule
						Longitudinal: no difference
				12 a*C9*+ vs. 50 NC	DTI	Baseline and follow-up: lower FA in frontotemporal tracts and higher MD in the entire skeleton
						Longitudinal: no difference

A few studies on familial FTLD have combined different mutation carriers as one group and compared them with non-carriers (Table 4). Combined asymptomatic *GRN* and *C9orf72* mutation carriers demonstrated overlapping atrophy in the insula (70), which has also been reported in a study combining asymptomatic *MAPT, GRN*, and *C9orf72* mutation carriers (6). No difference was observed in both cross-sectional (7) and longitudinal investigations (21) in the combined cohort of asymptomatic *MAPT* and *GRN* mutation carriers. However, in a small cohort of 5 *MAPT* mutation and 3 *GRN* mutation carriers who converted from asymptomatic to symptomatic phase, extensive temporal and frontal cortical atrophy were observed, while no gray matter volume loss was observed longitudinally in asymptomatic *MAPT* and *GRN* mutation carriers who remained asymptomatic compared to non-carriers (21).

**Table 4:** Studies investigating multiple different mutations in FTLD.

**No**.	**Author**	**Year**	**Study design**	**No. of subjects**	**Techniques**	**Findings**
1	Benussi et al. (70)	2019	Longitudinal	(48 a*GRN*+, 4 a*C9*+) vs. 73 NC	sMRI	GM volume loss in the insula
2	Jiskoot et al. (21)	2019	Longitudinal	35 non-converter (27 a*GRN*+, 8 a*MAPT*+) vs. 30 NC	sMRI	Non-converter: no difference at any time point
				8 converter (3 *GRN+*, 5 *MAPT+*) vs. 30 NC		Converter: GM volume loss (the prefrontal, temporal, cingulate, and insular cortex) from 2 years before symptom onset
					DTI	Non-converter: no difference at any time point
						Converter: extensive lower FA (the genu corpus callosum, forceps minor, uncinate fasciculus, and superior longitudinal fasciculus) from 2 years before symptom onset
3	Dopper et al. (7)	2014	Cross-sectional	39 carriers (28 a*GRN+*, 11 a*MAPT*+) vs. 36 NC	sMRI	No difference
					DTI	Reduced FA in the right uncinate fasciculus
					rfMRI	Reduced functional connectivity in the SN
4	Cash et al. (6)	2018	Cross-sectional	23 a*MAPT*+, 65 a*GRN*+, 40 a*C9*+ vs. 144 NC	sMRI	Atrophy in the anterior insula
5	Panman et al. (22)	2019	Longitudinal	14 a*MAPT*+ vs. 33 a*GRN*+	sMRI	Baseline: no differenceFollow-up: Cortical thinning in the right temporal pole in a*MAPT*+Longitudinal: no difference
				14 a*MAPT*+ vs. 28 a*GRN*+	DTI	No difference
6	Panman et al. (22)	2019	Longitudinal	11 a*C9*+ vs. 14 a*MAPT*+ (sMRI)	sMRI	Baseline and follow-up: lower GM volume in the cerebellum, thalamus, and insula in a*C9*+ Longitudinal: no difference
				12 a*C9*+ vs. 14 a*MAPT*+	DTI	No difference
7	Panman et al. (22)	2019	Longitudinal	11 a*C9*+ vs. 33 a*GRN*+	sMRI	Baseline and follow-up: lower GM volume in the cerebellum, thalamus, insula, and frontal cortical regions; and thinning cortical thickness in the precentral and postcentral gyrus in a*C9*+
						Longitudinal: no difference
				12 a*C9*+ vs. 28 a*GRN*+	DTI	No difference

*aMAPT+, asymptomatic MAPT mutation carriers; HC, healthy controls; PET, positron emission tomography; AChE, acetylcholinesterase; MRS, magnetic resonance spectroscopy; mI, myo-inositol; Cr, creatine; NAA, N-acetylaspartate; PCC, posterior cingulate cortex; NC, non-carriers; sMRI, structural MRI; rfMRI, resting state functional MRI; DMN =default mode network; DTI, diffusion tensor imaging; FA, fractional anisotropy; DR, radial diffusivity; ASL, arterial spin labeling; GM, gray matter; MD, mean diffusivity; WM, white matter; FDG, fluorodeoxyglucose; aGRN+, asymptomatic GRN mutation carriers; sym, symptomatic; EO, expected onset; aC9+, asymptomatic C9orf72 mutation carriers; SN, salience network*.

There has been a comparison of atrophy patterns resulting from mutations in different genes (22); the distinct pattern of atrophy in the early stage of disease course among the *MAPT, GRN*, and *C9orf72* mutation carriers align well with previous studies in symptomatic mutation carriers (67).

### Diffusion Tensor Imaging

Diffusion tensor imaging (DTI) is a sensitive technique to detect the white matter microstructure alterations. Damage to the white matter is a common finding in postmortem studies of sporadic and familial FTLD patients (71–73). Loss of white matter integrity on DTI was characterized by increased mean diffusivity (MD), radial diffusivity (RD), and axial diffusivity (AD), as well as reduced fractional anisotropy (FA).

#### *MAPT*_DTI

In asymptomatic *MAPT* mutation carriers, reduced FA and increased diffusivity were reported in bilateral uncinate fasciculus and cingulum in cross-sectional studies (7, 36), while a longitudinal study only found increased MD in entorhinal white matter with accelerated increase in MD during follow-up (38). Furthermore, in a longitudinal study, lower FA in uncinate fasciculus and other white matter tracts were reported in *MAPT* and *GRN* mutations 2 years before symptom onset, while no difference in FA was found between the combined group of asymptomatic *MAPT* and *GRN* mutation carriers who remained asymptomatic and non-carriers (21). Another recent longitudinal investigation also reported no difference in DTI measurements between asymptomatic *MAPT* mutation carriers and non-carriers (22).

Compared to FA, the diffusivity parameters such as MD appear to be more sensitive measures of the white matter integrity alterations in asymptomatic familial FTLD (36, 38). One possible explanation is that FA is more influenced by the crossing fibers, which may limit its sensitivity to detect subtle white matter changes (74). The underlying pathologic mechanism of white matter diffusion abnormalities in *MAPT* mutation carriers remains unclear. It may be due to the Wallerian degeneration secondary to the cortical tau pathology or to the tau pathology observed in the white matter. Future studies are needed to correlate these findings with pathology.

#### *GRN*_DTI

White matter involvement from DTI studies in asymptomatic *GRN* mutation carriers showed reduced FA in the left uncinate fasciculus, left inferior occipitofrontal fasciculus, and the genu of the corpus callosum (55). Reduced FA and increased diffusivity in the internal capsule was also reported (36). Another DTI investigation did not find a difference in FA, MD, and RD, but reported increased AD in the right cingulum, superior longitudinal fasciculus, and corticospinal tract (25). In a longitudinal design, reduced FA was found in bilateral superior longitudinal fasciculus, frontal corpus callosum, and left corticospinal tract in asymptomatic *GRN* mutation carriers, with greater annualized FA change in frontal corpus callosum and right superior longitudinal fasciculus compared to controls (27). Others did not report any abnormalities of DTI metrics in both cross-sectional (7) and longitudinal investigations (22) of asymptomatic *GRN* mutation carriers.

As indicated in sMRI, left–right asymmetry was also found in most white matter tracts of *GRN* mutation carriers. The most consistent asymmetry was reported in the uncinate fasciculus, retrolenticular part, and anterior limb of the internal capsule, as well as external capsule (36). However, the development of asymmetry over time may have different patterns in various white matter tracts. Longitudinal data with larger cohorts are needed for such investigations.

#### *C9orf72*_DTI

Investigations in asymptomatic *C9orf72* carriers with DTI have shown reduced white matter integrity in the fiber tracts connecting frontal lobe (the uncinate fasciculus and inferior longitudinal fasciculus), motor function-related tracts (corticospinal tracts, corona radiata, and internal/external capsule), thalamic radiation, as well as corpus callosum and cingulum bundles and cerebellar peduncles (30, 31, 36, 69). One cross-sectional investigation reported no DTI abnormality in asymptomatic *C9orf72* mutation carriers (29). A recent longitudinal investigation reported a more diffuse white matter involvement in asymptomatic *C9orf72* mutation carriers, with lower FA in the frontotemporal tracts and higher MD in the entire white matter skeleton compared to non-carriers. However, these white matter differences remained relatively stable during a 2-year follow-up period (22).

The heterogeneity in clinical phenotypes that ranged from motor neuron disease to FTD and the variation in time of symptom onset within and between families of *C9orf72* mutation carriers may be responsible for the variable results from cross-sectional DTI investigations in asymptomatic *C9orf72* mutation carriers. Longitudinal follow-up is essential in gaining insight into clinical and neuroimaging characteristics. Therefore, studying families with varying disease phenotypes along the ALS-FTD spectrum would be of particular interest in addressing the issue of clinical heterogeneity in relation to specific early neuroimaging changes.

### Functional MRI

Functional MRI can evaluate the alterations from functionally connected networks in spatially distinct subcortical and cortical areas in asymptomatic FTLD mutation carriers. In asymptomatic *MAPT* mutation carriers, resting-state fMRI demonstrated decreased connectivity in the default mode network (DMN) predominantly between lateral temporal lobe and precuneus (33). Components of the DMN, such as lateral temporal lobes and medial prefrontal cortex, were implicated in functions of semantic memory (75) and theory of mind (76), which show abnormalities in both patients with sporadic bvFTD and symptomatic *MAPT* mutation carriers. However, another fMRI study found no difference in functional connectivity between asymptomatic *MAPT* mutation carriers and controls (7). The change of functional connectivity was observed early in the disease course even without gray matter loss in asymptomatic *MAPT* mutation carriers (33), suggesting that functional abnormalities may precede the occurrence of atrophy in these regions.

In the asymptomatic *C9orf72* mutation carriers, the reduced functional connectivity was found prominently in salience network (SN), as well as in medial pulvinar of thalamus-seeded networks, DMN, and sensorimotor network (30). The fMRI alterations can already be distinguished in persons younger than 40 years of age (30). The SN is the main functional network associated with FTLD (77), which plays a role in behavioral functioning and emotion processing (78). Thus, reduced connectivity across central nodes of the SN may lead to some of the clinical features of FTLD.

Increasing evidence from the resting-state fMRI studies demonstrate widely affected networks in asymptomatic *GRN* mutation carriers, including both increased (56) and reduced connectivity in the SN (7), as well as reduced connectivity in the DMN (7). Functional activation of the SN was inversely related to global reserve index in asymptomatic *GRN* mutation carriers (60). Furthermore, hypoconnectivity of the left fronto-parietal network (FPN) and a hyper-connectivity within the executive network have been demonstrated (62). The functional impairment of parietal lobes had been thought to be the earliest feature of FTD with *GRN* mutations (61), while one study reported no difference in functional connectivity between asymptomatic *GRN* mutation carriers and non-carriers (25). When performing a relational reasoning task, asymptomatic *GRN* mutation carriers presented lower task-evoked functional activation in anterior and posterior ventrolateral prefrontal cortex compared to controls (64).

As suggested by theoretical concept of molecular nexopathies (79), a specific proteinopathy affects specific networks, while other brain networks could be dynamically involved during the course of the disease. This may possibly explain the heterogeneity in the fMRI findings in asymptomatic FTLD mutation carriers. However, other environmental or genetic factors can also affect the involved networks in a complex way. For example, TMEM106B genetic variations had a modulating effect on the functional connectivity in asymptomatic *GRN* mutation carriers. For example, the TT genotype of RMEM106B may cause additional neurodegeneration by reduced connectivity in the left fronto-parietal network and ventral SN (62). More studies with longitudinal follow-up are needed to further validate the use of functional connectivity as a potential biomarker for clinical trials.

### Proton Magnetic Resonance Spectroscopy

Proton magnetic resonance spectroscopy (^1^H MRS), which provides non-invasive measurements of brain biochemistry, is a potential imaging marker for early detection of neurodegenerative disease progression in familial FTLD. ^1^H MRS metabolite measurements have been sensitive biomarkers of early neurodegenerative pathology in FTLD, AD, and Lewy body dementia (80–85). *N*-acetylaspartate/Creatine (NAA/Cr) is considered a biomarker of neuronal integrity (86), neuronal viability (87, 88), and synaptic integrity (89), while elevated mI may be associated with astrocytic and microglial activation, as well as glial proliferation (90, 91).

In both asymptomatic and symptomatic *MAPT* mutation carriers, neurochemical alterations from the posterior cingulate voxel were found on single-voxel ^1^H MRS (92). An elevation in myoinositol (mI) or mI to creatine (mI/Cr) and a decrease in the neuronal integrity marker NAA or NAA/Cr have been found in symptomatic patients with FTLD (83), while only elevated mI/Cr in the posterior cingulate gyrus has been reported in asymptomatic *MAPT* mutation carriers (8). During longitudinal follow-up in *MAPT* mutation carriers who converted from the asymptomatic to symptomatic disease, serial ^1^H MRS from the posterior cingulate voxel demonstrated that metabolite ratio changes, characterized by increasing mI/Cr and decreasing NAA/mI ratios, begin to accelerate ~2 years prior to symptom onset (40).

However, ^1^H MRS from the medial frontal lobe voxel was more sensitive than ^1^H MRS from the PCC voxel in the asymptomatic *MAPT* mutation carriers, characterized by decreased NAA/Cr and NAA/mI ratios (39), suggesting specific regional involvement in neurodegenerative diseases. Regional differences that are apparent in the early stage of disease course may be lost as neurodegenerative pathology spreads to most of the brain regions during the later stages in *MAPT* mutation carriers.

Potentially, anterior temporal lobe ^1^H MRS measurements would be the most sensitive to early changes in *MAPT* mutation carriers. However, this may be offset by a reduction in the quality of the ^1^H MRS data, since anterior temporal lobes are proximal to the magnetic susceptibility artifacts from the skull base that impact the quality of the ^1^H MR spectra.

To date, no MRS study was reported in asymptomatic *GRN* and *C9orf72* mutation carriers. In the future, whole-brain and multi-voxel MRS could provide metabolite changes in more brain regions than single-voxel MRS (93). By utilizing more advanced acquisition methods, it would be possible to quantify metabolites, such as glutamine, glutathione, and Scyllo-Inosital (94). For example, an advanced ^1^H-MRS protocol composed of semi-localization by adiabatic selective refocusing (sLASER) localization and FAST(EST)MAP shimming detected lower glutamate concentration in patients with amnestic mild cognitive impairment than clinically normal controls indicating early Alzheimer's disease pathophysiology (95).

### Positron Emission Tomography

Positron emission tomography (PET) imaging is often suggested as a useful biomarker for the earliest stage of FTD and had demonstrated differences in metabolism by different tracers in the presymptomatic stage of familial FTLD.

#### *MAPT*_PET

In symptomatic *MAPT* mutation carriers, [^18^F]-fluorodeoxyglucose (FDG)-PET has showed asymmetric temporal lobe hypometabolism (46). Frontotemporal hypometabolism on FDG-PET was reported in two asymptomatic *MAPT* mutation carriers in the same family who had only mild speech change at the time of examination. These participants were in a transitional stage from normal neurological functioning to overt FTLD and one became symptomatic later, suggesting that FDG-PET changes can be detected when asymptomatic *MAPT* mutation carriers get close to phenoconversion. However, specificity of FDG-PET to differentiate between various neurodegenerative diseases at the early stages remains unknown (96).

Given the heterogeneity of tau pathology observed in *MAPT* mutations, tau PET (e.g., ^18^F-AV-1451 PET) was explored as a biomarker for various forms of tau pathology within a similar patient population. Subtypes of *MAPT* mutations were associated with different AV-1451 uptake patterns due to different types of underlying tauopathies. The mutations inside exon 10 (i.e., S305N, P301L, and N279K) with 4R tau pathology had a low level of AV-1451 binding in Tau PET in symptomatic *MAPT* mutation carriers. Mutations outside exon 10 (i.e., V337M and R406W) with mixed 3R/4R tau pathology that are more likely to produce AD-like tau pathology presented had a high magnitude of binding of AV-1451 in symptomatic *MAPT* mutation carriers (37). In symptomatic *MAPT* V337M mutation carriers, the tau accumulation assessed by [^18^F] AV1451 tracer was also associated with regional brain atrophy by structural MRI (97). Tau PET studies in asymptomatic *MAPT* mutation carriers are very limited. One asymptomatic *MAPT* N279K mutation carrier presented low level of AV-1451 uptake, one of two asymptomatic *MAPT* R406W mutation carriers had little to no signal and the other one had high level of AV-1451 uptake (37). General conclusions about the tau PET in asymptomatic *MAPT* mutation carriers remain inconclusive due to small number of cases reported so far.

Tracers associated with dopaminergic function were applied in *MAPT* mutation carriers, especially in the N279K mutation type, which may present with parkinsonism as the first symptom. Dopaminergic dysfunction was shown early in asymptomatic *MAPT* N279K mutation carriers via 2b-carbomethoxy-3b-(4-trmethylstannylphenyl) tropane (11C-CFT)-PET (34, 98). Glial activity was elevated in the frontal cortex, the posterior cingulate cortex, and the occipital cortex of asymptomatic *MAPT* mutation carriers on [^11^C] DAA1106 PET, and acetylcholinesterase activity was reduced in the temporo-parietal cortex using [^11^C] N-methylpiperidin-4-yl acetate PET in three asymptomatic *MAPT* mutation carriers (34). These case studies with small sample sizes will need to be replicated in larger cohorts.

#### *GRN*_PET

In asymptomatic *GRN* mutation carriers, hypometabolism was detected in right medial, ventral frontal cortex, and insula on FDG-PET (59). A longitudinal investigation reported that hypometabolism in the left middle temporal gyrus at baseline is associated with greater decrease in metabolism in the fronto-temporal lobes and thalamus in asymptomatic *GRN* mutation carriers compared to non-carriers (26).

#### *C9orf72*_PET

A radioligand for the microtubule associated protein tau (^18^F-Flortaucipir) was found to show increased binding in semantic variant primary progressive aphasia (svPPA) (99), which is associated with underlying TDP-43 pathology. In contrast, a recent study reported that none or limited ^18^F-Flortaucipir retention was found in symptomatic *C9orf72* mutation carriers, suggesting that ^18^F-Flortaucipir binding in svPPA patients is not a general TDP-43 related phenomenon (100). To date, a PET tracer for the *in vivo* detection of TDP-43 pathology does not exist.

## Conclusion

This review underscores the importance of imaging biomarkers for accurate prediction of symptom onset and tracking of disease progression during the presymptomatic stage of familial FTLD. The application of advanced neuroimaging techniques in monogenetic familial FTLD represents a unique model to detect the natural history of specific proteinopathies and clinical phenotypes. They may also provide a direct comparison across different gene groups in the future. Furthermore, multiple MRI and PET modalities may provide information on the different aspects and stages of the neurodegenerative disease process in a single individual. Many of the studies discussed in this review focus on a single imaging biomarker. Thus, it is not possible to compare biomarkers on their accuracy in prediction of onset of clinical symptoms and tracking disease progression in familial FTLD. The potential application of multi-model imaging is to provide evidence of very early asymptomatic alterations by collectively including cortical atrophy, white matter integrity loss, functional alteration, as well as brain metabolic changes. There are several ongoing prospective multisite studies (e.g., GENFI, ARTFL, and LEFFTDS) involving the familial FTLD kindred. These longitudinal investigations in large cohorts on the genotype-specific imaging profiles for *MAPT, GRN*, and *C9orf72* at different time points during the disease course would provide insights into their potential use as prognostic biomarkers for clinical trials of disease-modifying therapies.

## Author Contributions

QC data collection, analysis and interpretation of the data, drafting the manuscript. KK design or conceptualization of the study, data collection, analysis and interpretation of the data, drafting the manuscript.

### Conflict of Interest

The authors declare that the research was conducted in the absence of any commercial or financial relationships that could be construed as a potential conflict of interest.
